# Haptoglobin-Conjugated Gold Nanoclusters as a Nanoantibiotic to Combat Bacteremia

**DOI:** 10.3390/nano12203596

**Published:** 2022-10-13

**Authors:** Hsiu-Yi Chu, Lung-Ching Chen, Tsung-Rong Kuo, Chun-Che Shih, Sibidou Yougbaré, Yu-Han Chen, Tsai-Mu Cheng

**Affiliations:** 1 Graduate Institute for Translational Medicine, College of Medical Science and Technology, Taipei Medical University, Taipei 11031, Taiwan; 2 Graduate Institute of Biomedical Materials and Tissue Engineering, College of Biomedical Engineering, Taipei Medical University, Taipei 11031, Taiwan; 3 Division of Cardiology, Department of Internal Medicine, Shin Kong Wu Ho-Su Memorial Hospital, Taipei 111045, Taiwan; 4 International Ph.D. Program in Biomedical Engineering, College of Biomedical Engineering, Taipei Medical University, Taipei 11031, Taiwan; 5 Graduate Institute of Nanomedicine and Medical Engineering, College of Biomedical Engineering, Taipei Medical University, Taipei 11031, Taiwan; 6 Taipei Heart Institute, Taipei Medical University, Taipei 11031, Taiwan; 7 Division of Cardiovascular Surgery, Department of Surgery, Wan Fang Hospital, Taipei Medical University, Taipei 11696, Taiwan; 8 Institute of Clinical Medicine, National Yang Ming Chiao Tung University, Taipei 11230, Taiwan; 9 Department of Surgery, School of Medicine, College of Medicine, Taipei Medical University, Taipei 11031, Taiwan; 10 Institut de Recherche en Sciences de La Santé/Direction Régionale du Centre Ouest (IRSS/DRCO), Nanoro BP 218, 11, Burkina Faso

**Keywords:** gold nanoclusters, nanoantibiotics, haptoglobin, reactive oxygen species, antibacterial activity, hemoglobin, bacteremia

## Abstract

Gold nanoclusters have revealed great potential as nanoantibiotics due to their superior chemical and physical characteristics. In this study, a peptide with 83 amino acids derived from haptoglobin was utilized as a surface ligand to synthesize gold nanoclusters via a facile hydrothermal approach. Characterization of the structural and optical properties demonstrated the successful synthesis of derived haptoglobin-conjugated gold nanoclusters. The spherical derived haptoglobin-conjugated gold nanoclusters exhibited a (111) plane of cubic gold and an ultra-small size of 3.6 ± 0.1 nm. The optical properties such as ultraviolet-visible absorption spectra, X-ray photoelectron spectroscopy spectra, fluorescence spectra, and Fourier transform infrared spectra also validated the successful conjugation between the derived haptoglobin peptide and the gold nanoclusters surface. The antibacterial activity, reactive oxygen species production, and antibacterial mechanisms of derived haptoglobin-conjugated gold nanoclusters were confirmed by culturing the bacterium *Escherichia coli* with hemoglobin to simulate bacteremia. The surface ligand of the derived haptoglobin peptide of derived haptoglobin-conjugated gold nanoclusters was able to conjugate with hemoglobin to inhibit the growth of *Escherichia coli*. The derived haptoglobin-conjugated gold nanoclusters with an ultra-small size also induced reactive oxygen species production, which resulted in the death of *Escherichia coli*. The superior antibacterial activity of derived haptoglobin-conjugated gold nanoclusters can be attributed to the synergistic effect of the surface ligand of the derived haptoglobin peptide and the ultra-small size. Our work demonstrated derived haptoglobin-conjugated gold nanoclusters as a promising nanoantibiotic for combating bacteremia.

## 1. Introduction

Bacteremia is an infection of the blood by viable bacteria [1,2,3]. Asymptomatic bacteremia can be caused by normal daily behaviors such as conducting oral hygiene and after minor medical treatments [4]. Clinically benign infections in some people are temporary and have no significant sequelae. However, because of dysfunctional or overwhelmed immune responses, bacteremia can evolve into a bloodstream infection with serious clinical symptoms, including systemic inflammatory response syndrome, sepsis, septic shock, and multiple organ dysfunction syndrome [5,6,7,8,9,10,11]. With high mortality rates after the progression to sepsis, bacteremia requires urgent treatment via appropriate antibiotics. Bacteremia patients need administration of empiric antibiotic coverage to decrease morbidity and mortality [12,13,14,15,16,17]. In most cases, antibiotic therapy should be continued for 7~14 days [18]. Although antibiotic therapy is a common clinical treatment for bacteremia, developing alternative approaches to treat bacteremia is still an urgent task due to antibiotic resistance.

Recent developments in nanomaterials as nanoantibiotics have been validated to fight bacterial infections due to their extraordinary structural and optical properties [19,20,21,22,23,24,25,26,27]. Various types of nanomaterials, including metal/metal oxides, semiconductors, and polymers, have been explored as nanoantibiotics against bacteria [28,29,30,31,32,33,34,35]. For example, gold (Au) nanobipyramids with a (111) plane exhibited better photothermal performance than that of Au nanorods with a (100) plane for the effective photothermal killing of bacteria [36]. The metallic phase of 1T-MoS_2_ nanoflowers revealed photodynamic antibacterial activity due to their photoinduced reactive oxygen species (ROS) [37]. Under sunlight and near-infrared (NIR) irradiation, CuS nanosheets and CuS nanoparticles (NPs) produce photoinduced electrons which then react with atmospheric moisture to generate hydroxide and superoxide anion radicals and heat, resulting in antibacterial activity [38]. Gelatin-capped silver NPs were utilized as anti-infective therapeutics of *Staphylococcus aureus*-induced keratitis due to their strong ability to cripple the bacterial membrane potential and destroy bacterial membranes [39]. Great efforts have been devoted to developing versatile nanomaterials as nanoantibiotics for antibacterial applications. The in vitro and in vivo antibacterial activities of nanomaterials have shown promising potential for practical applications in the near future.

Among various nanomaterials, ultra-small metal nanoclusters (NCs) have been intensively demonstrated as nanoantibiotics to treat bacterial infections because of their physical and chemical characteristics [40,41,42,43,44,45]. Metal NCs with various metallic cores and surface ligands have been designed and proven to have antibacterial applications [46,47,48,49,50]. For instance, after being metabolized by *Escherichia coli*, cysteine-conjugated AuNCs generated significant intracellular ROS that induced the death of *E. coli* [51]. AuNCs conjugated with 6-mercaptohexanoic acid exhibited wide-spectrum antimicrobial activities for both gram-positive and gram-negative bacteria owing to their ultra-small size [52]. Metallic NCs such as Ag, Au, and Cu NCs modified with the surface ligand of bacitracin revealed robust bacteria-killing efficiencies because of their distinctive damage to bacterial membranes [53]. In real-time observations by liquid cell transmission electron microscopy (TEM), glutathione-capped AuNCs first attached to bacterial membranes and then penetrated into bacteria by internalization to cause destruction to the bacterial membranes, which led to the eventual death of the bacteria. Using combinations of metallic cores and surface ligands, metal NCs showed promising potential as nanoantibiotics based on their unique properties, including facile synthesis, easy surface modification, ultra-small size, and superior antibacterial activities.

A cell-free hemoglobin (Hb)-binding protein, haptoglobin (Hp), is an acute-phase protein in the blood that responds to bacterial infections and inflammation [54]. Hp plays an important role in protecting against bacterial infections due to its enhancement of bacterial growth by cell-free Hb [55]. Hp is currently being utilized as a therapeutic protein for bacteremia [56]. Herein, we cloned an anti-infection and anti-inflammatory multiple-functional peptide (MFP) with 83 amino acids derived from Hp that was applied as an antibacterial ligand to synthesize AuNCs (d-Hp-AuNCs), which were synthesized by a simple hydrothermal approach. The morphological and optical properties of d-Hp-AuNCs were investigated via TEM, high-resolution (HR)-TEM, energy-dispersive X-ray (EDX) spectroscopy, ultraviolet-visible (UV-Vis) spectroscopy, fluorescence spectroscopy, X-ray photoelectron spectroscopy (XPS), and Fourier transform infrared (FTIR) spectroscopy. To simulate bacteremia, the *E. coli* bacterium was cultured with Hb. To evaluate the antibacterial activity against bacteremia, the bacterial growth curve of *E. coli* incubated with d-Hp-AuNCs was examined. ROS generation by d-Hp-AuNCs incubated with *E. coli* was measured to investigate the antibacterial mechanism against bacteremia.

## 2. Materials and Methods

### 2.1. Materials

Sodium hydroxide (NaOH, 98%) and endotoxin removal spin columns were purchased from Thermo Fisher Scientific (Waltham, MA, USA). Tetrachloroauric(III) acid trihydrate (HAuCl_4_•3H_2_O, 99%) was purchased from Alfa Aesar (Haverhill, MA, USA). Tryptone, yeast extract, sodium chloride (NaCl), kanamycin, isopropyl β-D-1-thiogalactopyranoside (IPTG), DNase I, lysozyme, and ampicillin were purchased from BioShop (Burlington, ON, Canada). The proteinase inhibitor and Ni-NTA resin column were purchased from Sigma-Aldrich (St. Louis, MO, USA). Coomassie R-250 dye was purchased from AMRESCO (Solon, OH, USA). Amicon^®^ Ultra centrifugal filters (3K MWCO) were purchased from Merck Millipore (Burlington, MA, USA).

### 2.2. Synthesis of D-Hp-AuNCs via a Facile Hydrothermal Approach

d-Hp-AuNCs were synthesized by a facile hydrothermal approach according to the previous literature with some modifications [51]. An NaOH aqueous solution (1 M at 150 µL) was added to 1 mL of an HAuCl_4_ aqueous solution (25 mM). Afterward, 1 mL of the derived Hp peptide (3.014 mg/mL in phosphate-buffered saline (PBS) at pH 8.0) was introduced into the mixture containing NaOH and HAuCl_4_ and then stirred at 300 rpm and room temperature in a dark environment for 48 h. After 48 h, d-Hp-AuNCs had formed as a yellow-colored solution. The d-Hp-AuNC solution was then stored at 4 °C in a dark environment for further experiments.

### 2.3. Expression of the Derived Hp Peptide

The derived Hp peptide was expressed in *E. coli* BL21 with minor modifications. Herein, the pUC57-Amp vector was utilized to produce derived Hp peptide (Figure S1). *Escherichia coli* was cultured in 25 g of lysogeny broth (LB). LB was prepared by adding 10 g of tryptone, 5 g of yeast extract, 10 g of NaCl, and 50 mg of kanamycin to 1 L of distilled water under stirring at 180 rpm and 37 °C for 12 h. Afterward, the bacterial solution was re-inoculated with fresh culture medium and then incubated at 180 rpm and 37 °C for 4 h. After incubation, the optical density (OD) of the bacterial solution at 600 nm (OD600) was measured to be ~0.6. Expression of the derived Hp peptide was induced by adding 1 mM of IPTG at 16 °C for 16 h. Bacteria were harvested by centrifugation at 4 °C and 4000× *g* for 30 min, and then the bacterial pellet was stored at −20 °C for purification.

### 2.4. Purification of the Derived Hp Peptide

Bacterial pellets were re-suspended in 100 mL of lysis buffer (one tablet of proteinase inhibitor, 2 μg/mL DNase I, and 50 mg/mL lysozyme) under sonication at 4 °C. After sonication, lysates were centrifuged at 12,000× *g* and 4 °C for 30 min. Supernatants were filtered (through a pore size of 0.45 μm), and an Ni-NTA resin column (Sigma-Aldrich) was further used to purify the derived Hp peptide. Before purification, the Ni-NTA resin column was equilibrated with 10 mM of imidazole/PBS. After bonding to the Ni-NTA resin, the derived Hp peptide was eluted by a linear gradient of a 20 to 500 mM imidazole/PBS program. The eluted peptide was directly analyzed by sodium dodecylsulfate polyacrylamide gel electrophoresis (SDS-PAGE) and stained with Coomassie R-250 dye, as shown in the Supporting Information (Figure S2). The 3D structure of the derived Hp peptide is shown as Figure S3 in the Supporting Information. The derived Hp peptide was concentrated with Amicon^®^ Ultra-centrifugal filters (3K MWCO) and then dialyzed with PBS (at pH 8.0). After dialysis, the derived Hp peptide was immediately processed with endotoxin-removal spin columns and sterile filtration (0.22 μm) and stored at 4 °C for subsequent experiments.

### 2.5. Bacterial Growth Curve and Agar Plate Counts

The ampicillin-resistant *E. coli* (BL21DE3 strain) were applied for bacterial experiments. The *E. coli* culture medium was composed of 6 mL of LB medium and 100 μg/mL ampicillin. *Escherichia coli* was cultured in LB medium at 37 °C and 180 rpm for 16 h. After culturing for 16 h and to determine the bacterial curve, 1 μL of bacterial solution, 100 μL of LB medium, and 280 μg of Hb were first added to 96-well plates. Afterward, 5 μg of kanamycin, 50 μg of the derived Hp peptide, and 50 μg of d-Hp-AuNCs were added to each well, and then the 96-well plate was cultured at 37 °C in a shaker at 180 rpm. OD600 values of the wells were measured every 1 h for 8 h. The absorbance at 600 nm for each group of bacterial solution at 0 h was measured and set as 0. Afterward, the absorbance of each bacterial solution at 600 nm was measured every 1 h. To calculate the change in OD600 value, the absorbance of bacterial solution at 600 nm and 0 h was subtracted from the absorbance at 600 nm and different culture time. After incubation for 8 h, 10 μL of the suspension from each well was diluted by 10^4^-fold with LB medium. The diluted suspension (10 μL) was evenly spread onto an LB agar plate (LB with 15 g/L agar) and further incubated at 37 °C. After culturing for 24 h, colonies on the LB agar plate were counted.

### 2.6. Evaluation of ROS Production

2′,7′-Dichlorodihydrofluorescein diacetate (DCFDA) dye was applied to measure ROS generation. DCFDA can react with ROS to form 2′,7′-dichlorofluorescein (DCF). Thereafter, the DCF fluorescence at a wavelength of 525 nm as excited by a wavelength of 488 nm can be utilized to evaluate ROS generation. Furthermore, Hoechst 33342 dye with excitation/emission at 350/461 nm was used to calculate the total amount of *E. coli*. In this study, four different bacterial solutions were, respectively, prepared to measure ROS generation, including (i) *E. coli*, (ii) *E. coli* + Hb, (iii) *E. coli* + Hb + the derived Hp, and (iv) *E. coli* + Hb + d-Hp-AuNCs. The OD600 value of the *E. coli* solution was 0.3. Concentrations of Hb, derived Hp, and d-Hp-AuNCs were 2.8, 500, and 500 mg/mL, respectively. The four different bacterial solutions were incubated at 180 rpm and 37 °C for 4 h. After incubation for 4 h, DCFDA (10 μM) and Hoechst 33342 (1 μg/mL) were added to the four different bacterial solutions, and the solutions were incubated at 180 rpm and 37 °C for 30 min in the dark. Afterward, the four bacterial solutions were centrifuged at 10^4^ rpm for 2 min. Supernatants were carefully removed, and pellets were re-dissolved in 600 μL of sterilized water with vortexing. The fluorescence intensities of DCF and Hoechst 33342 were measured using a microplate reader. ROS production was calibrated with the total number of *E. coli*. Relative ROS levels of the experiments were systematically calculated compared to the ROS level of the control *E. coli* solution. For the control, the ROS level of *E. coli* incubated with sterilized water was set to 1.0.

### 2.7. Statistical Analysis

All experimental results were repeated four times. All the data and one-way ANOVA on Prism 9 (GraphPad Software, San Diego, CA, USA) were used to analyze the differences in significance between control and experimental groups. The numeric data are presented as the means ± standard deviation. A value of *p* < 0.05 was considered statistically significant.

## 3. Results and Discussion

### 3.1. Morphological Characteristics of D-Hp-AuNCs

**Figure 1:**
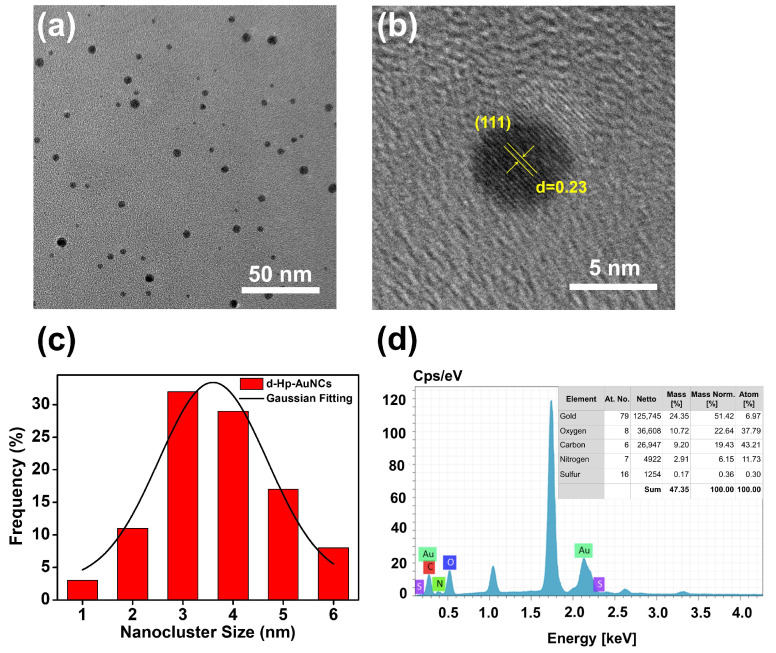
(**a**) TEM image of the derived haptoglobin peptide-conjugated gold nanoclusters (d-Hp-AuNCs), (**b**) HR-TEM image of d-Hp-AuNCs, (**c**) Histogram of the size distributions of d-Hp-AuNCs and the Gaussian fitting curve, and (**d**) EDX analysis of d-Hp-AuNCs.

To examine the morphology, d-Hp-AuNCs were characterized by TEM and HR-TEM. As shown in Figure 1a, d-Hp-AuNCs were homogeneously spread on a copper grid due to their conjugation with the derived Hp peptide to avoid aggregation. Furthermore, d-Hp-AuNCs revealed a roughly spherical shape. In the HR-TEM image of Figure 1b, d-Hp-AuNCs exhibited a (111) plane of cubic gold with an interplanar distance of 0.23 nm. To calculate the average size, size distributions of d-Hp-AuNCs were measured according to 100 NCs in Figure 1a, and the histogram is shown in Figure 1c. Based on the histogram of Figure 1c, the Gaussian fitting curve was simulated to obtain the average size of ultra-small d-Hp-AuNCs (3.6 ± 0.1 nm). Moreover, as shown in Figure 1d, the EDX analysis of d-Hp-AuNCs indicated that d-Hp-AuNCs were constituted of gold (51.42 wt%), oxygen (22.64 wt%), carbon (19.43 wt%), nitrogen (6.15 wt%), and sulfur (0.36 wt%). EDX measurements validated the composition of d-Hp-AuNCs with gold and the derived Hp peptide. To sum up, morphological characterizations demonstrated the successful preparation of d-Hp-AuNCs.

### 3.2. Optical Properties of D-Hp-AuNCs

**Figure 2:**
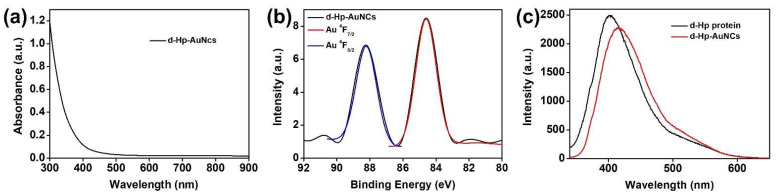
(**a**) UV-Vis absorption spectrum of the derived haptoglobin peptide-conjugated gold nanoclusters (d-Hp-AuNCs), (**b**) XPS spectra of d-Hp-AuNCs (black line), simulated spectrum of Au ^4^F_7/2_ (red line), and simulated spectrum of Au ^4^F_5/2_ (blue line). (**c**) Fluorescence spectrum of the derived Hp peptide (black line) and d-Hp-AuNCs (red line).

The absorption of d-Hp-AuNCs was first characterized by UV-Vis spectroscopy. In Figure 2a, the absorption of d-Hp-AuNCs exhibited no surface plasmon absorption of Au NPs at 520 nm [57]. The disappearance of surface plasmon absorption of Au NPs could be ascribed to the gold cores of d-Hp-AuNCs exhibiting high oxidation states, which resulted in a lack of free electrons to generate coherent oscillations [58]. To further demonstrate the high oxidation states of the gold cores, XPS was utilized to examine d-Hp-AuNCs. As shown in Figure 2b, the XPS spectrum of d-Hp-AuNCs indicated that the binding energies of Au ^4^F_5/2_ and Au ^4^F_7/2_ of d-Hp-AuNCs were 88.3 and 84.6 eV, respectively. The binding energies of simulated curves of Au ^4^F_5/2_ and Au ^4^F_7/2_ were also, respectively, located at 88.3 and 84.6 eV. On the other hand, the binding energies of Au ^4^F_5/2_ and Au ^4^F_7/2_ of bulk gold were separately revealed to be 87.4 and 84.0 eV. Compared to bulk gold, the higher binding energies of Au ^4^F_5/2_ and Au ^4^F_7/2_ of d-Hp-AuNCs indicated that the gold cores of d-Hp-AuNCs were composed of gold with high oxidation states, corresponding to the disappearance of surface plasmon absorption. Moreover, optical properties of d-Hp-AuNCs were examined via fluorescence spectroscopy. As shown in Figure 2c, under an excitation wavelength of 329 nm, the fluorescence spectrum of the derived Hp peptide exhibited a maximum fluorescence intensity at ~402 nm. For d-Hp-AuNCs, the maximum intensity was at ~415 nm. Compared to the fluorescence spectrum of the derived Hp peptide, the fluorescence of d-Hp-AuNCs was attributed to fluorescence coming from the conjugation of the derived Hp peptide. Overall, the optical properties, including the UV-Vis absorption spectra, XPS spectra, and fluorescence spectra validated the successful conjugation of d-Hp-AuNCs by a facile hydrothermal approach.

**Figure 3:**
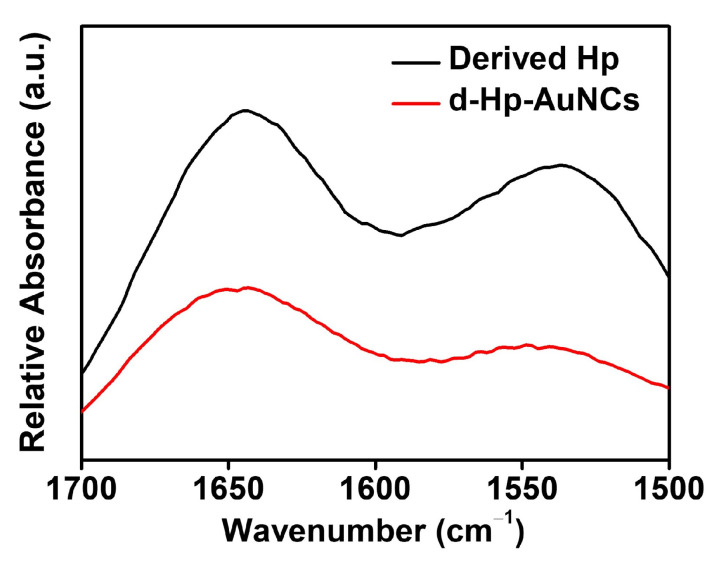
FTIR spectra of the derived haptoglobin (Hp) peptide and derived Hp-conjugated gold nanoclusters (d-Hp-AuNCs).

To further examine the conjugation between the derived Hp peptide and AuNCs, FTIR spectroscopy was applied to measure the IR bands of amide I and amide II of the derived Hp peptide and d-Hp-AuNCs. For the amide I band, the characteristic peak between 1600 and 1700 cm^−1^ was attributed to stretching vibrations of CO in the peptide. The characteristic peak of the amide II band between 1500 and 1600 cm^−1^ was attributed to NH bending and CN stretching vibrations within the peptide. In the FTIR spectra of Figure 3, the derived Hp peptide, respectively, revealed characteristic amide I and amide II peaks at 1644 and 1535 cm^−1^. d-Hp-AuNCs exhibited characteristic amide I and amide II peaks at 1644 and 1547 cm^−1^, respectively. Compared to the FTIR spectra of the derived Hp peptide and d-Hp-AuNCs, the amide I bands exhibited no significant change. However, the maximum absorption of the amide II band of d-Hp-AuNCs increased from 1535 to 1547cm^−1^ compared to that of the derived Hp peptide. The previous literature proved that the increase in the peak position of the amide II band can be ascribed to the formation of conjugation of the derived Hp peptide and AuNCs [59,60]. Overall, the optical properties, including the UV-Vis absorption spectra, XPS spectra, fluorescence spectra, and FTIR spectra validated the successful conjugation of d-Hp-AuNCs by a facile hydrothermal approach.

### 3.3. Antibacterial Activity of D-Hp-AuNCs

**Figure 4:**
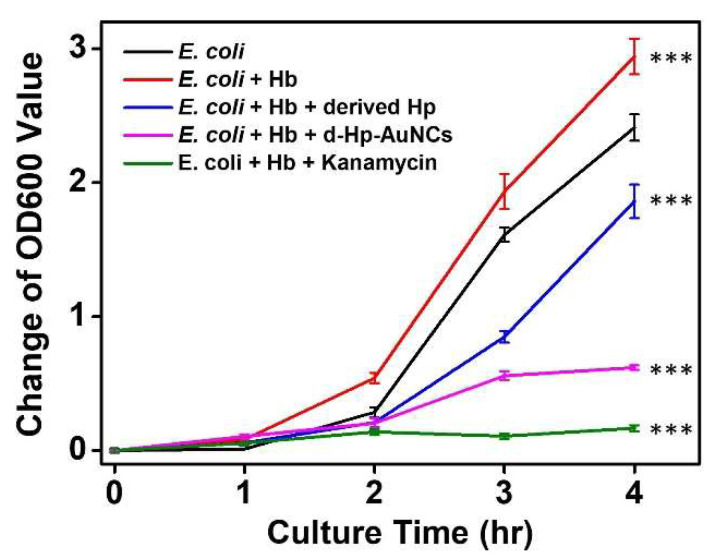
Growth curves of *Escherichia coli* incubated with various solutions, including (i) *E. coli,* (ii) *E. coli* + hemoglobin (Hb), (iii) *E. coli* + Hb + kanamycin, (iv) *E. coli* + Hb + derived haptoglobin (d-Hp), and (v) *E. coli* + Hb + d-Hp-gold nanoclusters (AuNCs). The results are presented as the mean ± SD of n = 4 independent experiments. Asterisks indicate significant differences (*** *p* < 0.001). *** *p* < 0.001 compared to *E. coli*.

To evaluate the antibacterial activities, five different bacterial solutions were prepared, including (i) *E. coli,* (ii) *E. coli* + Hb, (iii) *E. coli* + Hb + kanamycin, (iv) *E. coli* + Hb + derived Hp, and (v) *E. coli* + Hb + d-Hp-AuNCs. *Escherichia coli* was first cultured with Hb to simulate bacteremia. As shown in Figure 4, changes in the OD600 values of *E. coli* cultured with Hb were higher than those of *E. coli* cultured without Hb. Herein, Hb facilitated the growth of *E. coli*. The bactericidal antibiotic property of kanamycin was also applied to treat *E. coli* cultured with Hb. Kanamycin exhibited excellent antibacterial activity due to its suppression of bacteria synthesizing vital proteins [61]. Furthermore, compared to *E. coli* cultured with Hb, after incubation for 4 h, the OD600 value of *E. coli* cultured with Hb and the derived Hp decreased from 2.94 to 1.86. The antibacterial activity of the derived Hp can be attributed to its conjugation with Hb, which resulted in inhibition of bacterial growth [62]. Moreover, after incubation with d-Hp-AuNCs, growth of *E. coli* cultured with Hb was inhibited. Most importantly, the change in the OD600 value of *E. coli* cultured with Hb revealed no significant increase after incubation with d-Hp-AuNCs for 3 h. The result of antibacterial activity of d-Hp-AuNCs can be supposed by two hypotheses. First, the derived Hp peptide on the surface of d-Hp-AuNCs conjugated with Hb inhibited the growth of *E. coli*. Second, the ultra-small d-Hp-AuNCs induced ROS generation which caused the death of *E. coli*. With the synergistic effect of the surface ligand of the derived Hp peptide and ultra-small size, the d-Hp-AuNCs were demonstrated to be a promising nanoantibiotic.

**Figure 5:**
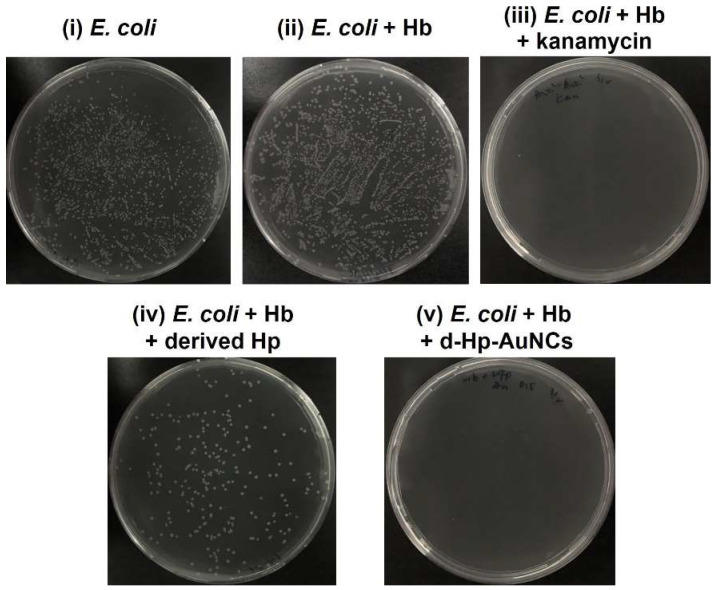
Photographs of the growth of *Escherichia coli* in five different bacterial solutions, including (**i**) *E. coli*, (**ii**) *E. coli* + hemoglobin (Hb), (**iii**) *E. coli* + Hb + kanamycin, (**iv**) *E. coli* + Hb + derived haptoglobin (d-Hp), and (**v**) *E. coli* + Hb + d-Hp-gold nanoclusters (AuNCs).

To evaluate the antibacterial activity, the five bacterial solutions, including (i) *E. coli*, (ii) *E. coli* + Hb, (iii) *E. coli* + Hb + kanamycin, (iv) *E. coli* + Hb + derived Hp, and (v) *E. coli* + Hb + d-Hp-AuNCs were investigated by agar plate counts after incubation for 4 h. As shown in Figure 5, colonies of (i) *E. coli*, (ii) *E. coli* + Hb, (iii) *E. coli* + Hb + kanamycin, (iv) *E. coli* + Hb + derived Hp, and (v) *E. coli* + Hb + d-Hp-AuNCs were, respectively, found to be 3.5 × 10^9^, 5.6 × 10^9^, 10^6^, 9.2 × 10^8^, and 0 colony-forming units (CFU)/mL. Furthermore, in the control experiment, the viability of (i) the *E. coli* solution was set to 100%. Viabilities of (ii) *E. coli* + Hb, (iii) *E. coli* + Hb + kanamycin, (iv) *E. coli* + Hb + derived Hp, and (v) *E. coli* + Hb + d-Hp-AuNCs were separately calculated to be 160%, 0.028%, 26%, and 0%. According to the results of agar plate counts, d-Hp-AuNCs exhibited outstanding antibacterial activity against *E. coli*. Overall, with the surface modification of the derived Hp peptide, the outstanding antibacterial activity of d-Hp-AuNCs was attributed to the synergistic effect of the derived Hp peptide and the ultra-small size.

### 3.4. ROS Generation by D-Hp-AuNCs

**Figure 6:**
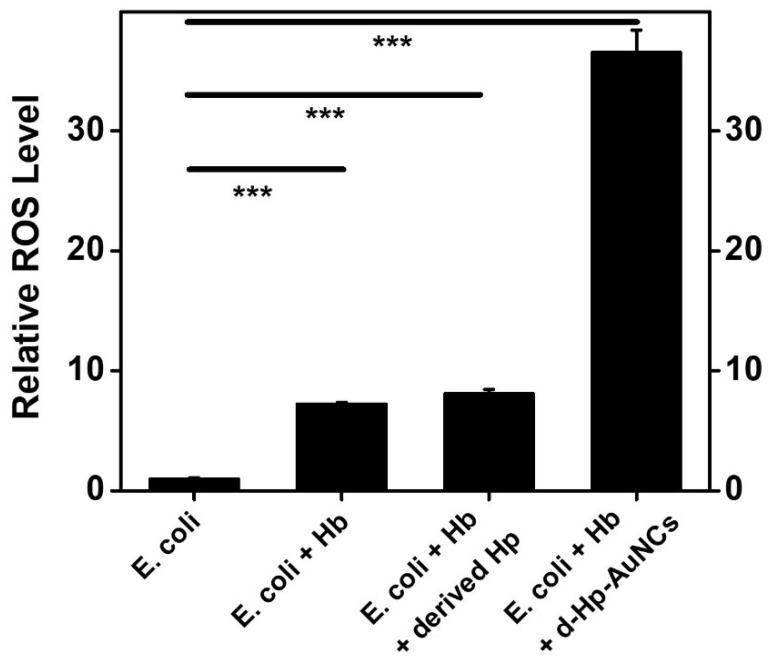
ROS levels measured with various solutions, including (i) *Escherichia coli*, (ii) *E. coli* + hemoglobin (Hb), (iii) *E. coli* + Hb + derived haptoglobin (d-Hp), and (iv) *E. coli* + Hb + d-Hp-gold nanoclusters (AuNCs). For the control, the ROS level of *E. coli* was set to 1.0. The results are presented as the mean ± SD of n = 4 independent experiments. Asterisks indicate significant differences (*** *p* < 0.001). *** *p* < 0.001 compared to *E. coli*.

A DCFDA assay was utilized to further investigate ROS generation by bacteria. Relative ROS levels of the four bacterial solutions of (i) *E. coli*, (ii) *E. coli* + Hb, (iii) *E. coli* + Hb + derived Hp, and (iv) *E. coli* + Hb + d-Hp-AuNCs were, respectively, 1.00-, 7.22-, 8.07-, and 36.53-fold higher, as shown in Figure 6. For solutions of (ii) *E. coli* + Hb and (iii) *E. coli* + Hb + derived Hp, there was only slight ROS production, corresponding to their bacterial growth curves. However, with incubation of Hb and d-Hp-AuNCs, the solution of *E. coli* exhibited remarkable ROS production. ROS generation induced by d-Hp-AuNCs can be attributed to the ultra-small size of the AuNCs because of better interactions with bacteria. With better interactions, ultra-small AuNCs could easily penetrate into bacteria, resulting in increased ROS that eventually killed the bacteria. Overall, the results of ROS measurements indicated that ultra-small d-Hp-AuNCs with superior antibacterial activity revealed great potential as a nanoantibiotic to treat bacteremia in the near future.

## 4. Conclusions

The derived Hp peptide was utilized as a surface ligand to synthesize d-Hp-AuNCs by a hydrothermal approach. The structural and optical characterizations, including TEM images, HR-TEM images, EDX analysis, UV-Vis spectra, XPS spectra, fluorescence spectra, and FTIR spectra, demonstrated the successful preparation of d-Hp-AuNCs. Bacterial growth curves, agar plate counts, and ROS production were investigated to demonstrate the antibacterial activity and mechanisms of d-Hp-AuNCs against *E. coli* cultured with Hb. The antibacterial activity of d-Hp-AuNCs was confirmed because the derived Hp peptide on the surface of the d-Hp-AuNCs was conjugated with Hb to inhibit the growth of *E. coli,* and the ultra-small d-Hp-AuNCs induced ROS generation to cause the death of *E. coli* [63]. Furthermore, smaller d-Hp-AuNCs (average size of ~3.6 nm) induced higher ROS production compared to that of bigger cysteine-conjugated gold nanoclusters (average size of ~4.1 nm) [51]. Based on the synergistic effect of the surface ligand of the derived Hp peptide and ultra-small size, the d-Hp-AuNCs exhibited excellent antibacterial activity against bacteremia. Our findings demonstrated that d-Hp-AuNCs could be developed as a potential nanoantibiotic for combating bacteremia.

## Data Availability

The data presented in this study are available on request from the corresponding author.

## Supplementary Materials

The following supporting information can be downloaded at: https://www.mdpi.com/article/10.3390/nano12203596/s1, Figure S1. The plasmid map of pUC57-Amp vector. Figure S2. The SDS-PAGE showed the fraction of purified MFP. Figure S3. The 3D structure of the derived Hp peptide.
