# Supplementary material for: Haptoglobin-Conjugated Gold Nanoclusters as a Nanoantibiotic to Combat Bacteremia
Source: Nanomaterials (Basel). 2022 Oct 13;12(20):3596. doi: 10.3390/nano12203596 (PMC9611519; doi:10.3390/nano12203596)
Supplement: Supplementary file 1 [file nanomaterials-12-03596-s001.zip › nanomaterials-1913445-supplementary.pdf]

nanomaterials

Supplementary Materials

Haptoglobin-Conjugated Gold Nanoclusters as a Nanoantibiotic to Combat Bacteremia

Hsiu-Yi Chu [1,2,†], Lung-Ching Chen [3,†], Tsung-Rong Kuo [4,5,†], Chun-Che Shih [6,7,8,9], Sibidou Yougbaré [10], Yu-Han Chen [1] and Tsai-Mu Cheng [1,6,*]

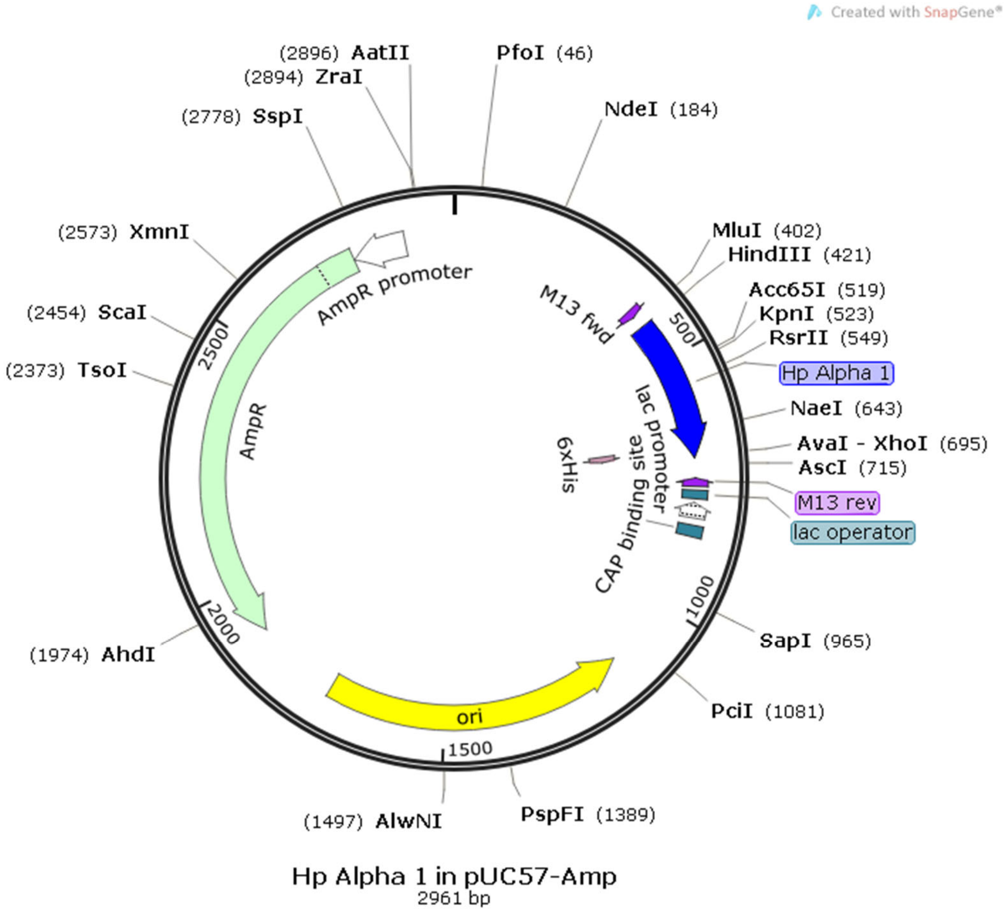

Figure S1. The plasmid map of pUC57-Amp vector.

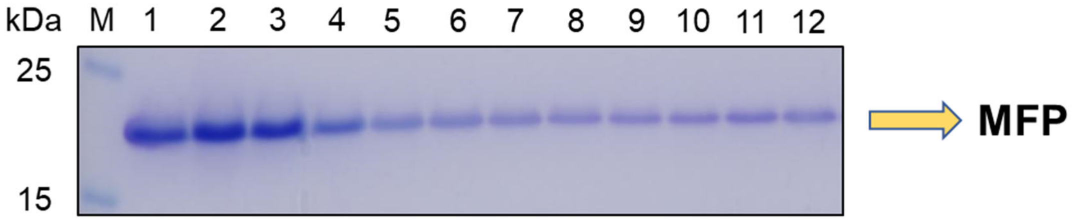

Figure S2. The SDS-PAGE showed the fraction of purified MFP. MFP expressed in *E. coli* was purified by His-tag affinity column. The fractions of eluent were applied to run SDS-PAGE as visualized by Coomassie R-250 staining. The purity >95% eluted fractions were pulled and further concentrated in PBS (pH 8.0) for experiments. M: MW marker, 1-12: Elution fraction, MFP: multiple functional peptide.

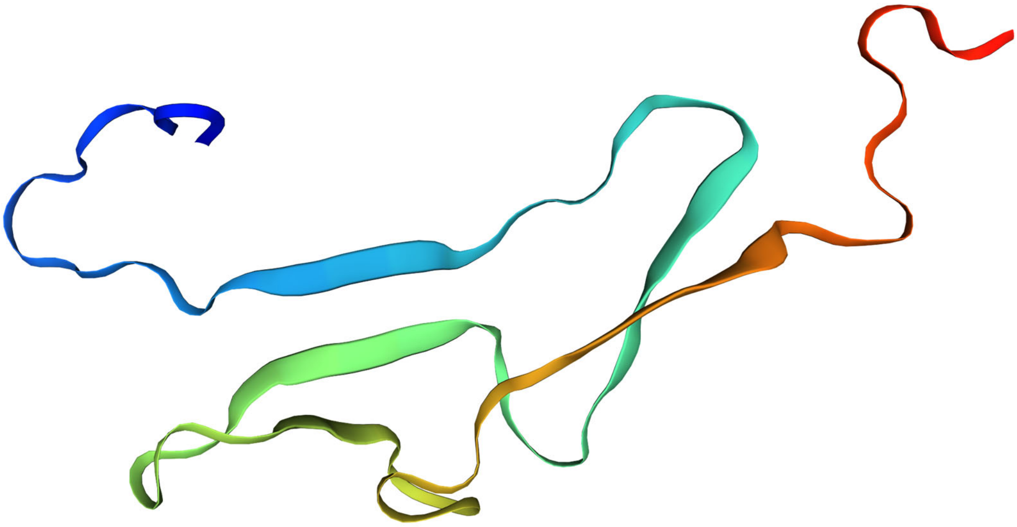

Figure S3. The 3D structure of the derived Hp peptide.
